# New Pipeline for Analysing Fruit Proteolytic Products Used as Digestive Health Nutraceuticals

**DOI:** 10.3390/ijms251910315

**Published:** 2024-09-25

**Authors:** Iván Benito-Vázquez, Ana Muñoz-Labrador, Manuel Garrido-Romero, Gema Hontoria-Caballo, Carlos García-García, Marina Diez-Municio, F. Javier Moreno

**Affiliations:** 1 Instituto de Investigación en Ciencias de la Alimentación, CIAL (CSIC-UAM), Nicolás Cabrera 9, 28049 Madrid, Spain; ivan.benito@pharmactive.eu (I.B.-V.); ana.munoz@csic.es (A.M.-L.); manuel.garrido@pharmactive.eu (M.G.-R.); 2 Pharmactive Biotech Products SLU, Faraday 7, 28049 Madrid, Spain; ghontoria@pharmactive.eu; 3 Centro de Biología Molecular Severo Ochoa, CBM (CSIC-UAM), Nicolás Cabrera, 1, 28049 Madrid, Spain; cgarcia@cbm.csic.es

**Keywords:** proteolytic products, nutraceutical, cysteine proteases, caseinolytic activity, digestive aid

## Abstract

Proteolytic products are extensively used in the nutraceutical sector to improve protein digestion and muscle quality in target populations (e.g., athletes or elderly). These products are processed using techniques that often lead to low purity but competitive pricing. Despite their widespread use and well-established production methods, the industry lacks standardized analytical methods for assessing these products and detecting potential fraud. This study proposes a comprehensive and harmonized pipeline for their analysis, which includes quantifying total soluble protein and proteolytic activity, as well as the determination of product stability and protein profile using SDS-PAGE and proteomic techniques. Despite the fact that protease extracts from pineapple had the highest protein content, most of the bromelain remained inactive, unlike in kiwi and papaya. SDS-PAGE revealed partial protein degradation of pineapple extracts, whereas kiwi extracts reflected a lower purification level but a higher protein integrity. The application of proteomic approaches strengthened the identification and origin tracing of the proteases. This study contributes to the development of a robust framework for analyzing proteolytic extracts, spanning from soluble protein quantification to protein profiling and activity determination. It may also ensure reliable supplier selection, high-quality manufacturing practices, and the implementation of optimal storage and formulation strategies in the nutraceutical industry.

## 1. Introduction

There is a growing awareness among specific populations aiming to improve protein digestion of protein-rich diets and to enhance muscle quality, such as among those at risk of developing sarcopenia or athletes with muscle injuries, who frequently consume digestive aid enzymes, mainly proteases. This scenario predicts a substantial increase in the use of enzyme supplements and digestive aids in the future. In that sense, there is a rising trend towards the industrial production of plant-based enzymes due to consumer acceptance and demand. To meet this demand and surpass the established enzyme production from microbial sources, it is crucial to improve the consistency and knowledge of the composition, structure, and enzyme activities of commercial plant-based protease products. Several issues in commercial plant-based protease supplements need to be addressed to enhance their industrial production and application. These limitations include a lack of homogeneity in the standardization determining specific enzyme activity, with inconsistencies in the substrates used and in methods to measure the proteolytic activity, as well as structural and compositional knowledge of the protein profile, in order to avoid fraud and the selling of products as highly-purified enzymes when they are just concentrated-fruit products.

Diseases like sarcopenia are highly influenced by poor nutrient absorption and/or low protein digestion. This disease is particularly prevalent in the elderly population and is characterized by a reduction in skeletal muscle mass below a critical level, which can result in functional impairment and physical disability [1]. Within this framework, various supplements (e.g., proteins, amino acids, or peptides) or nutritional strategies have been suggested to enhance muscle mass and prevent muscle atrophy [2]. One of these is related to enhancing the absorption of these compounds with proteolytic extracts [3]. In fact, Oben et al. demonstrated that there was an increase in the absorption rate of processed whey protein concentrate when a patented blend of digestive proteases was introduced into the human diet [4]. This approach could also aid in reducing the dose of protein required for muscle recovery or stimulation. It may also be particularly beneficial for high-level athletes who typically consume protein above the daily recommended allowance due to their high energy-demanding lifestyle [5]. Additionally, these kinds of products are beneficial for athletes aiming to enhance muscle quality and growth, helping to reduce the risk of muscle injuries associated with high-performance sports [6].

Among digestive enzymes, microbial proteases have traditionally been the most extensively studied enzymes [7] and they are used in a wide range of industries [8,9]. They are generally derived from bacteria that have undergone genetic modifications [10,11,12]. Despite their proven safety, this aspect may lead to a lower acceptance among consumers of the nutraceuticals developed using these proteases, as they might be mistakenly perceived as genetically modified organisms or transgenics foods [13,14,15]. In that sense, plant cysteine proteases, like actinidin, bromelain, papain, or ficin, have gained attention in recent years due to the rising demand for products derived from natural sources and due to their diversity, enhanced specificity, and the availability of straightforward and environmentally friendly processes for their production [16,17]. Cysteine proteases catalyze the hydrolysis of peptide, amine, ester, thiol ester, and thiono ester bonds [18].

For instance, bromelain (EC 3.4.22.33) and papain (3.4.22.2) enzymes are the most well-studied and used cysteine proteases in the pharmaceutical, nutraceutical, and food industries [18,19,20]. Papain can alter gastric motility, which could explain its claimed beneficial effects in preventing functional gastrointestinal disorders [21], reducing inflammation and helping in the amelioration of obesity [22]. Instead, the bromelain spectrum includes acting as a reversible inhibitor of platelet aggregation, alleviating sinusitis, aiding in recovery from surgical traumas, combating bronchitis, and facilitating the enhanced absorption of drugs, particularly antibiotics. Additionally, bromelain exerts anti-inflammatory and anti-microbial properties [23,24]. On the other hand, actinidin (EC 3.4.22.14) and ficin (3.4.22.3) enzymes are two cysteine proteases that have been increasingly popular in the last few years due to their growing importance in the food and biotechnology industries. Ficin is extracted from the latex of fig trees (*Ficus carica*) and is widely known for the tenderization of meat targeting collagen and elastin, enhancing its texture and its moisture retention [25,26,27]. Alternatively, actinidin, an enzyme present in kiwi fruit (*Actinidia deliciosa*), has potential as a digestive aid for humans and animals [28], improving protein digestion and gastric emptying [29,30]. It is also widely used in meat tenderization [31] and texture and stability improvement of dairy products [32], showcasing its versatile applications beyond its digestive benefits.

As mentioned above, despite this industrial interest, there is a lack of consistency in the analyses of commercially available proteolytic products derived from fruits. The great variety of substrates to quantify proteolytic activity in these kinds of products, like Lys-oNP [33], azocasein [34], casein [35], or gelatin [36] hinder an adequate comparison among commercial products. Furthermore, the specific enzyme activity is not provided and only the activity per gram is normally disclosed by the nutraceutical industry. This determination may not accurately reflect the product’s quality, potentially including a significant amount of non-enzymatic protein content. Additionally, comprehensive characterization of proteolytic products derived from fruits in the literature is limited [37,38], being more pronounced in the case of actinidin products from kiwifruit. Even so, simple analyses like soluble protein content are not shown in the labelling of these kinds of products. Frequently, quality control laboratories that purchase these products are unsure of which analyses to perform in their laboratories to verify the product’s integrity, to detect the presence of other enzymes, to infer purity, or to accurately compare enzymatic activity according to their final application [37].

This study proposes an assessment for the first time of the protein content, enzymatic activity, SDS-PAGE, and proteomics analyses across a great variety of fruit-derived commercial products available in the nutraceutical industry. Such a harmonized pipeline is suggested for regular adoption in the nutraceutical industry. This comprehensive analysis will facilitate the identification of reliable suppliers and ensure the high-quality of manufacturing processes, apart from offering a useful resource to improve awareness in the nutraceuticals market regarding the characterization of proteolytic assays. Moreover, it should assist in determining optimal storage conditions and formulating the final product effectively.

## 2. Results

### 2.1. Biochemical Properties

#### 2.1.1. Total Soluble Protein

**Figure 1:**
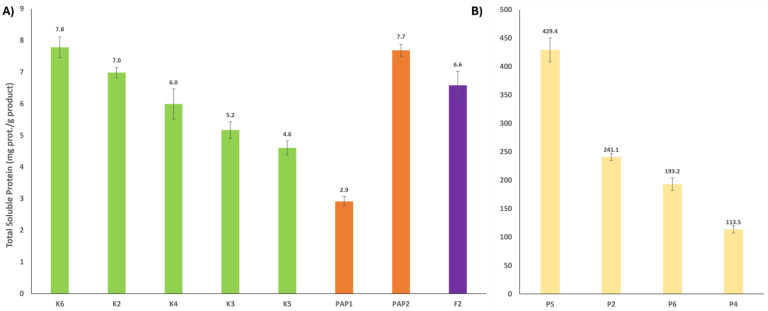
Total soluble protein in milligrams per gram of commercial product. (**A**) K, kiwi products; PAP, papaya products; P, pineapple products; and (**B**) F, Fig products.

The total soluble protein per gram of assessed commercial PPs is shown in Figure 1. Pineapple products (Figure 1B) exhibited nearly 40-fold higher protein content per gram on average compared to kiwi, fig, and papaya products (Figure 1A) (*p* < 0.001). Even within the pineapple samples, there were significant differences, with P5 having by far the highest protein content (429.4 mg/g).

In contrast, slight differences were found among the kiwi samples: K6 and K2 had the significantly highest protein content (7.8 and 7.0 mg/g), (*p* < 0.029), while K3 and K5 had the lowest protein content (5.2 and 4.6 mg/g), respectively. K4 had intermediate values with a slight yet significant difference compared to K2 and K3, *p* < 0.004, and *p* < 0.022. Among papaya products, PAP1 had, remarkably, the lowest protein content (2.9 mg/g) compared to the entire sample set, while PAP2 (7.7 mg/g) was located between the K6 and K2 samples.

Based on the proposed characterization of PPs, K6, PAP2, and P5 have emerged as the preferred products, given that a significant portion of the protein content is likely to be attributable to enzymes. Regarding the F2 sample, the unique sample available in the market, its enzymatic activity might be compared with kiwi and papaya products due to its relatively high protein content and anticipated enzyme activity. In the absence of significant differences between kiwi and papaya products, the industry would prioritize net value as the primary decision-making factor, considering these differences as secondary even when they are not statistically significant.

#### 2.1.2. Proteolytic Activity

**Figure 2:**
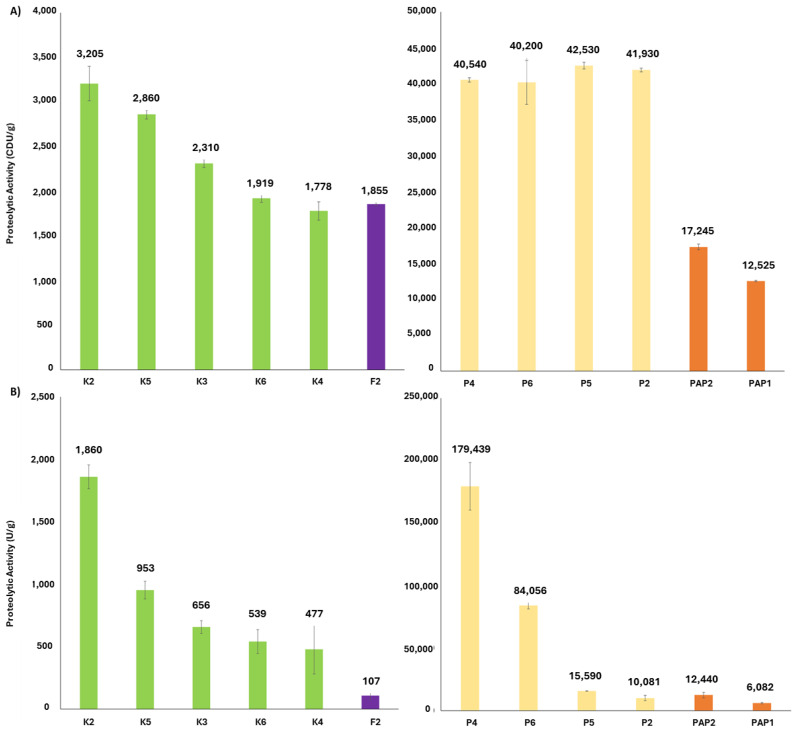
Proteolytic activity using as a substrate casein (**A**) or Z-L-Lys-ONp hydrochloride (**B**). K, kiwi products; PAP, papaya products; P, pineapple products; and F, Fig products. CDU, Casein Digestion Units. U, units.

Two types of proteolytic assays based on the substrate used, i.e., casein (Figure 2A) and Z-L-Lys-ONp hydrochloride, were carried out to determine the proteolytic activity (Figure 2B). The product distribution obtained from these proteolytic assays differed significantly from that of the total soluble protein.

In the casein assays, pineapple products were the most active, showing no significant differences among them and averaging 40,000 CDU/g—40 times higher than kiwi products and 2.5 times higher than papaya products on average. Papaya products were the second most active, with PAP2 exhibiting the slightly highest activity (*p* < 0.007). Among the kiwi products, K2 and K5, displayed nearly the same activity, both higher than the other kiwi products, which formed a single group without significant differences. The fig product, which had one of the lowest activities, was also part of this group.

On the other hand, in the Lys-ONp assays, differences in the activity of pineapple products were even more pronounced, being 80 and 5 times higher on average than kiwi and papaya products, respectively. Among the pineapple products, P4 and P6 stood out significantly, while P2 and P5 had the lowest activity with no difference between them. Similarly, within the papaya products, PAP2 was again the most active. For kiwi products, only K2 showed a significant difference compared to the others, which formed a unique group with no differences among them, including the fig product, which had the lowest proteolytic activity. As in the casein assays, K2 was the most active in its group.

**Figure 3:**
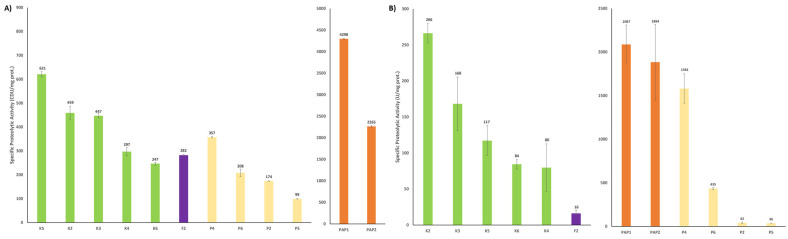
Specific proteolytic activity of fruit products using as a substrate casein (**A**) or Z-L-Lys-ONp hydrochloride (**B**). K, kiwi products; PAP, papaya products; P, pineapple products; and F, Fig products. CDU, Casein Digestion Units. U, units.

Figure 3 shows the specific proteolytic activity for casein (A) and Lys-ONp (B) assays, which quantifies the amount of proteolytic active enzymes present in the whole extract. Using this criterion as a benchmark, papaya products exhibited the highest quantity of active papain per milligram of protein in both analyses (1834 to 4293 U/mg). The only exception was P4 in the Lys-ONp assay, which showed identical specific proteolytic activity to PAP1 and PAP2, with no significant differences between them. This fact contrasts to the casein assay, where PAP1 showed a higher value compared to PAP2.

Based on the specific proteolytic activity, pineapple products had lower activity compared to all the other extracts, indicating that most of the protein present in these products is not active bromelain. In the casein assay, pineapple products exhibited the same or even lower activity than the majority of kiwi products and the fig. A similar trend was observed in the Lys-ONp assay with P2, P5, K4, K5, and K6 with no difference among them in contrast to CDU/g (Figure 3A) and U/g (Figure 3B) representations. According to our data, the best product would be PAP1, K2/K5, and P4.

#### 2.1.3. Enzyme Stability

**Figure 4:**
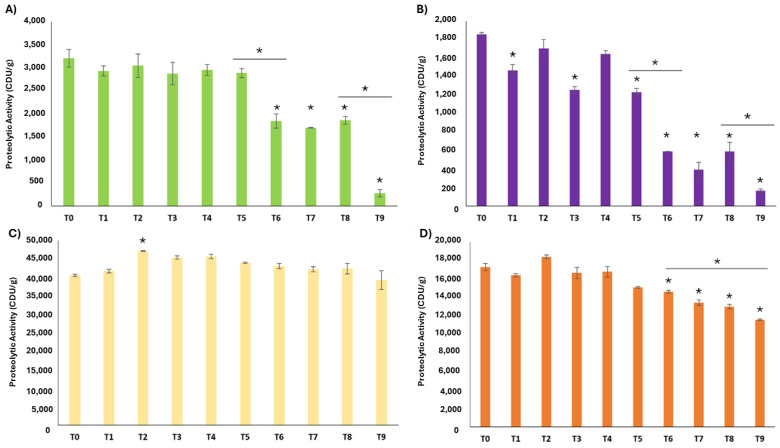
Stability test in long-term conditions (25 °C, 60% relative humidity) of fruit proteolytic products (PPs) using casein assay during 6 months for K2 (**A**), F2 (**B**), P4 (**C**), and PAP2 (**D**). T1, 1 week. T2, 2 weeks. T3, 3 weeks. T4, 1 month. T5, 1.5 months. T6, 2 months. T7, 2.5 months. T8, 3 months. T9, 6 months. * *p* < 0.001, significant difference with respect to T0. Horizontal bar to compare bars at both ends.

The stability test was conducted based on the casein (Figure 4) and Lys-ONp (Supplementary Figure S2) results, from which one fruit extract from each PP group was selected. Among kiwi products, K2 was selected due to its highest activity within the group in both proteolytic assays and its specific proteolytic activity in the Lys-ONp assay. In the pineapple group, P4 was chosen due to its high overall activity performance in both proteolytic activity tests, including its specific activity. PAP2 was chosen as the representative for the papaya group because it had the highest net proteolytic activity in both analyses, the most valued measure in the industry, despite having the lowest specific activity in the casein assay. Finally, F2 is the only fig product available for the stability test. Generally, all fruit PP extracts maintained their proteolytic activity for, at least, 1.5 months (T5) according to both proteolytic activity tests (Figure 4 and Supplementary Figure S2).

For caseinolytic activity, K2 (Figure 4A) kept its activity for 1.5 months (T5) under long-term conditions. Subsequently, there was a significant decrease in activity over the next two months (T6), followed by stabilizing up to the timepoint of three months (T8). After T8, at 6 months (T9), K2 caseinolytic activity dropped to near zero. Similarly, F2 (Figure 4B) showed stable activity for 1.5 months (T5), with a decline that persisted until three months (T8), and nearly disappeared by six months (T9). Although T1, T3, and T5 were significantly lower than T0, they seemed to be outliers because there was not a downward trend through time until T6. For P4 (Figure 4C), there was a significant increase in the enzymatic activity from the initial point (T0) to 2 weeks (T2), followed by a slight but not significant decrease up to T9 (6 months). Finally, PAP2 activity began to decrease at 1.5 months (T5) and showed no significant differences thereafter until 6 months (T9) of storage.

Conversely, in the Lys-ONp assay (Figure S2), the stability test variability was higher and the trends over time were harder to discern. In fact, there were no significant differences among different time points for any commercial product except F2, where the activity ceased at 2 months (T6).

### 2.2. Product Origin and Protein Profile Determination

#### 2.2.1. SDS-PAGE

**Figure 5:**
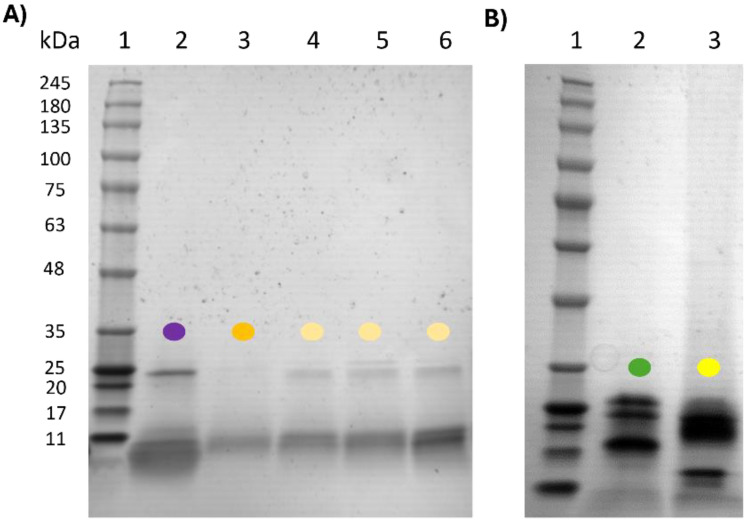
SDS-PAGE fruit controls. (**A**) Lane 1. Protein Marker. Lane 2, Fig. Lane 3, Papaya. Lane 4, Pineapple Pulp. Lane 5, Pineapple Core. Lane 6, Pineapple Peel. (**B**) Lane 1, Protein Marker. Lane 2, Kiwi Green. Lane 3, Kiwi Gold. Code of colors: Purple, fig. Light yellow, pineapple. Bright yellow, kiwi gold. Green, kiwi green. Orange, papaya.

SDS-PAGE analyses were performed to investigate the origin, qualitative purity, and enzyme/protein integrity of the commercial PPs, as well as the samples resulting from the extraction of the original fruits (i.e., in-house extracts) used as positive controls. As shown in Figure 5, the band patterns ranged from 11 to 245 kDa for the in-house PPs. For fig and papaya samples (Figure 5A), a relative molecular mass (Mr) of 23 kDa band was identified, which could tentatively correspond to ficin (with an estimated Mw range of 22–30 kDa) [39,40] and papain [41], respectively. These bands were faint due to the fact that these enzymes are normally extracted from fig and papaya latex, and the whole fruit preparations used in this study have low protein concentrations. Pineapple lanes (Figure 5A) equally displayed apparent bands with Mr around 26 and 23 kDa, which presumably belong to core and pulp bromelain, respectively [38].

In contrast, the kiwi green and gold preparations (Figure 5B) displayed four and three main proteins in their electrophoretic profiles, respectively. For the kiwi green sample, bands appeared at Mr 26.5, 23.3, 17.4, and 14 kDa, tentatively corresponding to actinidin, thaumatin-like protein (TLP), KiTH, and kirola (also known as Act d 11), respectively. In kiwi gold, the identified proteins were at 22.8, 20.3, and 14 kDa, which are likely to correspond to actinidin, an actinidin isoform, and kirola, according to the bibliography [42].

**Figure 6:**
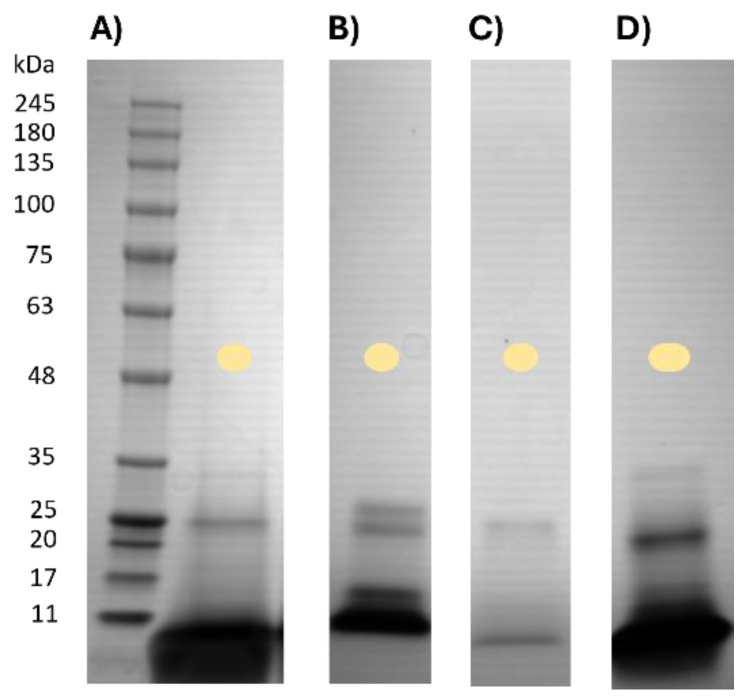
SDS-PAGE pineapple products. (**A**) Protein Marker and pineapple product P4. (**B**) Pineapple product P5. (**C**) Pineapple product P2. (**D**) Pineapple product P6. Code of colors: Light yellow, pineapple.

Figure 6 illustrates the electrophoretic profiles of various commercial pineapple products, wherein certain samples required a prior concentration step to observe the complete protein profile. P5 exhibited two bands at Mr of 26.5 and 23 kDa, in agreement with the pineapple protein profile attributed to core and pulp bromelain, respectively. In the case of P6 (Figure 6D), a single protein around 25 kDa, which is similar to core bromelain found in P2 (Figure 6C), was observed. In contrast, P4 (Figure 6A) showed an additional band at approximately 31 kDa, potentially indicating a post-translationally modified bromelain similar to P6, which falls within the range of 20 to 31 kDa as previously reported [43].

It is noteworthy that pineapple products contained a significant quantity of low molecular weight proteins. Despite this, the electrophoretic profile of P5 (Figure 6B) closely resembled that of the fruit preparation, while P2, P4, and P6 shared at least one common band.

**Figure 7:**
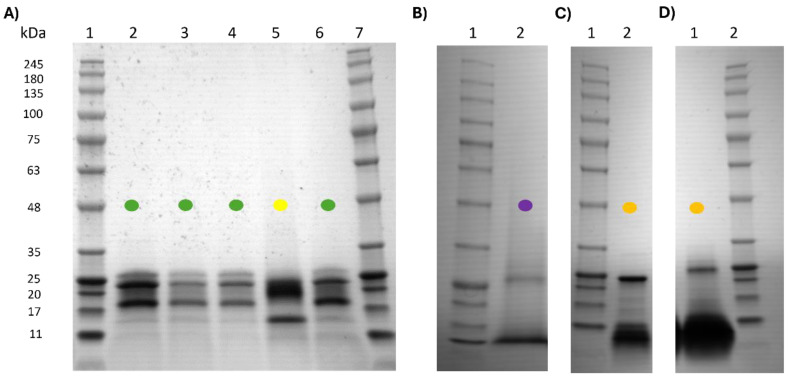
(**A**) Kiwi products SDS-PAGE. Lane 1 and 7, Protein Marker. Lane 2, Kiwi Product K2. Lane 3, Kiwi Product K4. Lane 4, Kiwi Product K3. Lane 5, Kiwi Product K5. Lane 6, Kiwi Product K6. (**B**) Fig product SDS-PAGE. Lane 1, Protein Marker. Lane 2, Fig product F2. (**C**) SDS-PAGE Papaya product. Lane 1, Protein Marker. Lane 2, Papaya product PAP1. (**D**) SDS-PAGE Papaya product. Lane 1, Papaya product PAP2. Lane 2, Protein Marker.

The electrophoretic profiles of commercial kiwi products (Figure 7A) exhibited similar patterns for both kiwi green and gold variants, although there were variations in the intensity of protein bands between them. In contrast, the fig product (Figure 7B) displayed a band with a Mr of 26 kDa, slightly higher than that of the fruit, yet within the range of 22–30 kDa reported for different ficin isoforms in the literature.

For papaya products, the electrophoretic profiles were consistent across samples. The most notable finding was in the PAP1 product, which exhibited bands at approximately 24, 18, 15, and 12 kDa. The band around 24 kDa tentatively corresponded to papain (approximately 23 kDa), as indicated in the literature [41]. The electrophoretic profiles of fig and papaya products closely resembled those of their respective fruit preparations. While fig products displayed a single band, albeit with a different molecular weight, papaya products exhibited additional bands, particularly in PAP1, although they shared the characteristic band of Mr around 23–24 kDa corresponding to papain.

Furthermore, like pineapple products, papaya products showed a significant amount of low molecular weight proteins, in contrast to those from kiwi and fig.

#### 2.2.2. Protein Identification by LC-MS^2^-Based Proteomics

To complement the SDS-PAGE analysis, K2, F2, PAP2, and P4 were selected for LC-MS^2^-based proteomics analysis following the same criteria as in the enzyme stability studies, whereas P5 and K5 were also selected to collect further information about other protein profiles different for the same family of proteolytic products.

As shown in Table 1, kiwi products had the most comprehensive identified protein composition compared to the rest of the studied fruit PPs. Overall, this finding is in line with the results shown by the SDS-PAGE analyses, where kiwi products had the highest number of bands in the electrophoretic profile, while all other extracts had 1, 2, or 3 main visualized bands. This may also highlight the degree of purification of the enzymes alongside this analysis, because the greater the variability of protein species detected from the fruit species, the lower the degree of purification, which could be indicative of a less elaborate production process. Consequently, this could also imply that there could be higher protease contents relative to total proteins in the papaya, pineapple, and fig products as compared to the extracts derived from kiwi.

**Figure 8:**
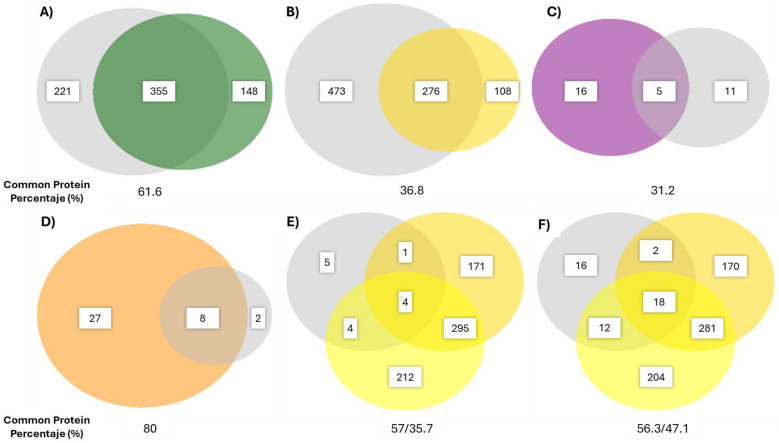
Venn diagrams of identified protein of fruit (colored) and commercial products (grey) using proteomics. (**A**) Kiwi green. (**B**) Kiwi gold. (**C**) Fig. (**D**) Papaya. (**E**) Pineapple P4 (dark-yellow pineapple core and light-yellow pineapple pulp). (**F**) Pineapple P5 (dark-yellow pineapple core and light-yellow pineapple pulp). The common protein percentage in P4 and P5 is divided into pineapple pulp and pineapple core by a slash.

In this context, since an identical proteome was utilized for analysing both the fruit in-house and the commercial products, their comparability was ensured. The quantification was determined by a common protein percentage, defined as the proportion of proteins identified in the database in both types of samples relative to the total number of proteins identified in the commercial product (Figure 8). These values could potentially highlight the origin of the product and provide insights into the purity and the process used to obtain the corresponding products. Thus, PAP2 seemed to be the most similar product compared to its origin fruit with an overlap of 80%, followed by K2 (61.6%), P4/P5 (57/56.3%) compared to pineapple pulp, (35.7/41.7%) to pineapple core, K5 (36.8%), and F2 (31.2%).

Table 2 discloses the cysteine proteases and other main proteins identified by LC-MS^2^-based proteomics and that were tentatively identified by SDS-PAGE analysis. Thus, actinidin was identified in kiwi green and K2, whose theoretical Mw (27.5 kDa) matched with the Mr (26.5 kDa) previously observed by SDS-PAGE. Actinidin was also present in kiwi gold and K5 according to proteomics data, although it could not be previously identified by SDS-PAGE. This discrepancy could be explained by the fact that LC-MS^2^ exhibits a higher sensitivity and power resolution than SDS-PAGE. Actinidin-like protein, which might be an isoform, was present in kiwi gold and K5, displaying a similar theoretical Mw (23.3 kDa) and Mr as determined by SDS-PAGE (22.8 kDa) [44]. Thaumatin-like protein (TLP), Kiwellin, KiTH-2 like protein, and Kirolla were identified in kiwi green, kiwi gold, and their respective commercial products K2 and K5. It is noteworthy that Kiwellin and TLP could not be distinguished by SDS-PAGE due to their similar electrophoretic mobility. Kiwellin can be cleaved in four proteins, kissper, KiTH-1, KiTH-2, KiTH-3 whose theoretical Mw are 4.2, 15.6, 15.4, 15.8 kDa, respectively [44]. Proteomics data only confirmed the presence of KiTH-2 like protein (Table 1) in the analysed samples.

In the case of fig samples, proteomics analysis of ficin confirmed the presence of up to 8 different isoforms/variants of ficin, whose amino acid sequences differed only in a few amino acids according to the theoretical Mw (Figure S3A,B).

Similarly, proteomics analysis of papaya samples showed different isoforms or redundant entries of different cysteine proteases, confirming the presence of papain, which was previously tentatively identified by SDS-PAGE. Whereas only two bands at 26 and 23 kDa could be visualized by SDS-PAGE, more comprehensive proteomics data were obtained from pineapple in-house extract and its commercial products, which served to confirm the presence of the two different cysteine proteases bromelain and ananain [45], apparently with the same theoretical Mw. No post-translational modifications (i.e., glycosylation) were detected by proteomics.

## 3. Discussion

The rising incidence of human gastrointestinal disorders [46], the demand for enhancing muscle quality in targeted populations, such as athletes and those vulnerable to sarcopenia, along with contemporary consumer preferences for natural and plant-based products, all foresee a high consumption of plant-derived enzymes as supplements and digestive aids in the future. In order to strengthen the commercial viability of these type of products and surpass the traditional protease production from microbial sources, it is critical to improve the consistency of composition, structure, and enzyme activities of commercial plant-based protease extracts through standardized protocols, including the harmonization of reference substrates and methodologies [47].

In this regard, there is a plethora of analytical methods available for the characterization of protease extracts, ranging from measuring total protein using either Kjeldahl, Bradford, or BCA, to proteomics-based approaches. During the measurement of total soluble protein content by the Bradford method (Figure 1), we could highlight the huge difference among these kinds of products, not commonly described all together in the bibliography, pineapple having the most soluble protein content compared to the rest. Although this parameter could seem irrelevant, it is very useful to obtain specific activity (Figure 3) and to determine the integrity of the product, autolysis, or storage degradation by quantifying the amount of protein as active enzyme. In addition, it is an affordable process for small companies in combination with proteolytic activity measurement. The quantification of the proteolytic activity of commercial products is very diverse, but two different substrates are normally used: casein [48], and Lys-oNP (N2-[(phenylmethoxy)carbonyl]-L-lysine 4-nitrophenyl ester hydrochloride) [34,49,50,51]. All these assays offer enzyme units that are not directly comparable among them, the first one the most popular considering its feasibility, simplicity and cost. Indeed, this method is widely used to determine proteolytic activity in bromelain and actinidin products through an L-Tyrosine calibration curve. Using the optimal conditions, e.g., temperature and pH during a certain time of reaction, the absorbance, measured at 280 nm, increases according to the liberation of peptides with aromatic amino acids derived from casein hydrolysis [35,52,53,54]. In this work, we used this method to measure the proteolytic activity (Figure 2A), the pineapple products being the most active. However, taking into account the ratio caseinolytic activity/total soluble protein through specific activity, these products would be less effective than kiwi products (Figure 3), highlighting the importance of this parameter.

On the other hand, the Lys-oNP method is based on the capacity of proteases to hydrolyse the Lys-oNP peptide bond with the liberation of the colour compound *p*-nitroanilin [32,55,56]. Nevertheless, the broken bond is an ester bond, not a peptide bond like those present in the casein during the caseinolytic assay. For this reason, this assay has been criticized for quantifying the proteolytic activity and, consequently, other substrates, like real proteins (i.e., casein) have been alternatively proposed to evaluate the final effect of the product [37]. Moreover, we have demonstrated that the variability of Lys-oNP assay makes the control of the product during the stability tests unreliable (Supplementary Figure S2), the caseinolytic assay being the best option, not only because of its close approximation to the real final product’s application but also because of its higher reliability.

Finally, SDS-PAGE [37] and proteomics [57] have been used to deeply analyse proteolytic commercial products in order to characterize the protein profile. Through SDS-PAGE analysis, we could show how the origin of the products rightly corresponded to the product’s description by comparing the protein profiles (Figure 5, Figure 6 and Figure 7). However, in some cases, we could elucidate the short-step purification process in the kiwi protein profile of commercial PPs, being fairly similar to that of freeze-dried pulp (Figure 5 and Figure 6), where other protein bands than actinidin appeared. In contrast, for fig products, we only observed a single band, which is supposed to be ficin (Figure 5 and Figure 6), despite that the control’s production was even less complex than that for kiwi. Thus, the nature of the fruit should be considered, since kiwi is richer in proteins than mature whole fig. In addition, to assess the purification grade of the product, we should know about the fruit protein characteristics and its stage of ripening, highly important for the protein content and profile. Similarly, papaya (Figure 6) and pineapple (Figure 7) products seemed highly purified, but their production process is quite simple and the whole parts of the fruit already showed simple protein profiles (Figure 5). Therefore, the production process would only affect the concentration of the enzyme and not the protein profile. SDS-PAGE could determine the origin of the products and, tentatively, the purity of the product if the knowledge of the raw material is available, as well as the presence of other proteins or different profiles in the case of potential frauds.

As could be expected, proteomics (Table 2) is a more reliable technique to detect other exogenous proteins and determine the presence of the expected enzymes. We accurately demonstrated the origin of the product and confirmed the absence of other enzymes in all products, asserting the low fraud rate in the tested samples. Using proteomics, a parameter that we have called common protein percentage (Figure 8) has been calculated. This parameter requires further in-depth study, as it will depend, as previously mentioned, on the ripeness and variety used of the reference fruit, and the production process employed. This variability makes it difficult to establish a numerical criterion for assessing a product’s origin. Nevertheless, the protein profile determined by SDS-PAGE and a thorough understanding of the raw material make its identification possible, as demonstrated in our results. (Figure 8). However, it is an expensive technique, which can be used in cases of difficult-to-resolve issues for medium and large-sized companies.

## 4. Conclusions

In this work, we have developed a straightforward pipeline for analyzing these products, quantifying total soluble protein and proteolytic activity, assessing product stability, and profiling proteins with SDS-PAGE and proteomic-based methods. Furthermore, employing each methodology outlined in this flowchart has enabled a suitable comparison of enzyme efficiency and performance among the tested commercial plant-based protease products derived from different sources, as well as their protein identification. Thus, papaya-derived products exhibited the highest specific protease activity, followed by kiwi proteolytic extracts, whereas pineapple products showed the lowest activity (Figure 3), despite accounting for the highest total protein content. Regarding the comparison of the two proteolytic assays based on different targeted substrates, i.e., casein and Z-L-Lys-ONp hydrochloride, the use of the former led to a higher reproducibility and less variability in the determination of the proteolytic activity of the commercial products. Additionally, since these products are marketed as protein digestion boosters, proteolytic activity should be measured using a natural protein rather than a synthetic substrate. This approach provides a more accurate approximation of the enzyme’s effect in the stomach [37]. Both enzyme assays were utilized in stability tests in long-term conditions at 25 °C and 60% relative humidity, showcasing their utility based on laboratory resources and objectives. Interestingly, all fruit enzymatic extracts kept their proteolytic activity unaltered for at least 45 days of storage.

Protein profiling data confirmed the partial degradation in extracts from pineapple probably due to auto-proteolysis, the low degree of purification of the commercial products derived from kiwi, as well as the presence of the main cysteine proteases (i.e., papain, actinidin, ficin, bromelain and ananain) in both the fruit in-house extracts and commercial samples. As expected, SDS-PAGE presented limitations in the unambiguous protein identification of the origin of cysteine proteases as they exhibited a similar size (around 20–30 kDa), preventing the unravelling of some potential frauds by, for instance, mixing cysteine proteases from different sources unnoticed in the labelling. However, proteomics-based approaches based on LC-MS shed light on the comprehensive characterization of the extracts’ composition, since proteins were more precisely identified, thereby demonstrating purity and aiding in fraud detection.

## 5. Materials and Methods

### 5.1. Fruit In-House Extracts and Commercial Samples

Commercial proteolytic products (PPs) were sourced from various distributors across Asia, Europe, and Oceania (Table 3). To produce the PPs’ in-house extracts, the reference fruits were separated into parts and the enzyme-rich fractions were crushed and lyophilized. The process for kiwi fruits involved peeling, crushing, and separation of the seeds by centrifugation (Mega Star 1.6/1.6R, VWR, Radnor, PA, USA) at 4000 rpm for 10 min, followed by freeze-drying of the pulp (Heto PowerDry LL1500 Freeze Dryer, ThermoFisher, Waltham, MA, USA) [58]. For fig and papaya samples, the entire fruit was crushed and freeze-dried. Finally, the core and pulp of the pineapple sample were manually prepared by crushing them separately and freeze-drying.

### 5.2. Total Protein Quantification

Protein concentration was determined by dissolving PP powders in water, centrifugating at 10,000 rpm for 5 min, and then the supernatant was subjected to the Bradford assay [59]. Briefly, 250 µL of each sample was mixed with 5 µL of Bradford reagent (Merck, Darmstadt, Germany) and incubated at room temperature for 5 min. After that, the absorbance was measured at 595 nm using an Epoch^TM^ spectrophotometer (Bioteck, Winooski, VT, USA). Bovine serum albumin (BSA) (Merck, Darmstadt, Germany) was used as a standard to generate a calibration curve (0.1–1.4 mg/mL). All samples and standards were assayed in triplicate to ensure the accuracy and reproducibility of the results.

### 5.3. Proteolytic Assays

#### 5.3.1. Casein Assay

Proteolytic activity using casein as a substrate was performed in triplicate using the assay based on the methods of Kunitz et al. and Tello-Solís et al. [35,60] with some modifications. Enzymes from PPs were extracted from 50–100 mg weighted in 10 mL of phosphate buffer 0.1 M, pH 6.0, 5 mM ethylenediaminetetraacetic acid (EDTA, Merck, Darmstadt, Germany), and 5 mM cysteine (Merck, Darmstadt, Germany). After that, PP solutions were mixed at a 1:1 ratio with 2% (*w*/*w*) sodium caseinate (Merck, Darmstadt, Germany) in phosphate buffer 0.1 M, pH 6.0, and incubated at 40 °C for 10 min. The reaction was stopped by the addition of 2 volumes of trichloroacetic acid (TCA) (Merck, Darmstadt, Germany) 5% (*v*/*v*) for 30 min in order to precipitate all undegraded proteins. The reaction mixture was filtered using a nylon filter 25 mm, 0.45 µm (Agilent, Santa Clara, CA, USA). The absorbance of the filtered solution was measured at 280 nm in a UV-visible spectrophotometer Genesys 50 (Thermo Fisher Scientific, Waltham, MA, USA) and the proteolytic activity was expressed as the amount of enzyme that releases 1 µg of tyrosine per minute under the assay conditions [61]. The blank solution was prepared by subjecting a casein solution to the same reaction conditions, halting the reaction with 5% TCA, adding the PP solutions, and then filtering the mixture after 30 min. One casein digestion unit (CDU) was defined as the amount of enzyme for 1 µg of tyrosine per minute. Supplementary Figure S1A shows the tyrosine calibration curve used for CDU determination.

#### 5.3.2. Lys-oNP Assay

Z-Lys-ONp hydrochloride (Lys-oNP; > 98%) (Merck, Darmstadt, Alemania) was used as a substrate to quantify the proteolytic activity of PP solutions at 0.1–0.005 mg/mL, with assays conducted in triplicate following the method described by Baker et al. and Bouland et al. with some modifications [62,63]. The reaction mixture consisted of 10 µL of Lys-oNP at 20 mM in methanol mixed with 990 µL of PP solutions in phosphate buffer 0.1 M, pH 6.0, 5 mM cysteine and EDTA at 25 °C. A UV-visible spectrophotometer was used to follow the 4-nitrophenol (4-NP) liberation at 400 nm for 5 min. The blank consisted of the reaction mixture prepared with 0.1 M phosphate buffer, pH 6.0, 5 mM cysteine, and EDTA under the described conditions. One unit (U) of activity was defined as the amount of enzyme that liberates 1 µmol of 4-NP per minute. Figure S1B,C show the resulting 4-NP calibration curves.

### 5.4. Stability Assay

Selected PP samples were subjected to long-term storage conditions at 25 °C at 60% relative humidity (RH). Samples were taken at 1, 2, 3 weeks, 1, 1.5, 2, 2.5, 3, and 6 months. Proteolytic activity was measured using both caseinolytic and Lys-oNP assays as explained in Section 5.3.

### 5.5. Sodium Dodecyl Sulfate-Polyacrylamide Gel Electrophoresis (SDS-PAGE)

Soluble proteins quantified by Bradford present in PP solutions were subjected to SDS-PAGE. PP solutions containing about 10 µg of protein were diluted at a 1:1 ratio with 2X Laemmli sample buffer (Bio-Rad, Hercules, CA, USA) containing 2-mercaptoethanol (Thermo Fisher Scientific, Waltham, MA, USA) and heated at 100 °C for 5 min. SDS-PAGE was performed using a MiniProtean Tetra Cell (Bio-Rad, Hercules, CA, USA) on a NZY Tris-Glycine Precast Gel (4–15%, 12 wells) (NZY, Lisbon, Portugal) with a Tris/glycine/SDS buffer system. Afterwards, the gels were stained with Coomassie blue (Merck, Darmstadt, Germany). In the case of pineapple, bands were not initially detected by SDS-PAGE and Amicon^®^ (Merck, Darmstadt, Germany) MWCO 10 kDa-ultrafilters were used to concentrate bromelain.

### 5.6. Proteomics

#### 5.6.1. Protein Digestion

The protein extracts were resuspended in a volume of 50 µL of sample buffer and then applied into an SDS-PAGE gel (0.75 mm thick, 4% stacking and 10% resolving). The unseparated protein bands were visualized by Coomassie staining, excised, cut into cubes (2 × 2 mm) and placed in 0.5 mL microcentrifuge tubes. The gel pieces were destained in acetonitrile/water (1:1; v:v) to remove the Coomassie blue and to initiate the dehydration of the dyed bands. Next, they were reduced with 10 mM DTT for 1 h at 56 °C and alkylated with 10 mM iodoacetamide for 30 min at room temperature in darkness and digested in situ with sequencing grade trypsin (Promega, Madison, WI, USA) as described by Shevchenko et al. with minor modifications [64]. The gel pieces were shrunk by removing all liquid using acetonitrile and the gel pieces were dried in a SpeedVac concentrator (Thermo Fisher Scientific, Waltham, MA, USA) after removing all the acetonitrile. The dried gel pieces were reswollen in 100 mM Tris-HCL pH 8, 10 mM CaCl_2_ with 60 ng/µL trypsin at a protein/enzyme ratio of 5:1 (*w*/*w*). The tubes were kept in ice for 2 h and incubated at 37 °C for 12 h and the digestion was stopped by the addition of 1% TFA. Whole supernatants were dried down and then desalted onto OMIX Pipette tips C18 (Agilent Technologies) until the mass spectrometric analysis.

#### 5.6.2. Reversed-Phase Liquid Chromatography–Mass Spectrometry (RP-LC–MS/MS) Analysis (Dynamic Exclusion Mode)

The desalted protein digest was dried and resuspended in 10 µL of 0.1% formic acid and analyzed by RP-LC–MS/MS in an Easy-nLC II/1200 system coupled to an ion trap LTQ-Orbitrap-Velos-Pro hybrid mass spectrometer (ThermoFisher, Waltham, MA, USA)). The peptides were concentrated by reverse phase chromatography using a 0.1 mm × 20 mm C18 RP precolumn (ThermoFisher, Waltham, MA, USA) and then separated using a 0.075 mm × 250 mm bioZen 2.6 mm Peptide XB-C18 RP column (Phenomenex) operating at 0.25 µL/min.

Peptides were eluted using a 90-min dual gradient as follows: 5–25% solvent B for 68 min, 25–40% solvent B for 22 min, 40–100% solvent B for 2 min, and 100% solvent B for the final 18 min. Solvent A consisted of 0.1% formic acid in water, while solvent B was composed of 0.1% formic acid and 80% acetonitrile in water. ESI ionization was performed using a Nanobore emitter (Stainless Steel ID 30 µm, Proxeon) at a spray voltage of 2.1 kV, with the S-Lens set to 60%. The Orbitrap resolution was configured to 30,000 [65].

Peptides were detected in survey scans from 400 to 1600 amu (1 μscan), followed by twenty data-dependent MS/MS scans (Top 20), using an isolation width of 2 u (in mass-to-charge ratio units), normalized collision energy of 35%, and dynamic exclusion applied during 60-s periods. Charge-state screening was enabled to reject unassigned and singly charged protonated ions.

#### 5.6.3. Data Processing

Peptide identification from raw data was carried out using PEAKS Studio v11.5 search engine (Bioinformatics Solutions Inc., Waterloo, ON, Canada). Database search was performed against uniprot-Ananas-comosus.fasta for pineapple (23,408 entries; UniProt release February 2024), uniprot-Rosales for fig (74,6021 entries; UniProt release 02/2024), uniprot-Brassicales for papaya (131,0247 entries; UniProt release February 2024) and uniprot-Actinidia-chinensis for kiwi green/gold (32,913 entries, UniProt release February 2024).

In the case of kiwi, the reference proteome available was *Actinidia chinensis*, which was used for the two kiwi species analyzed. For pineapple, its species-specific reference proteome was used. For papaya and fig, reference proteomes used were from their closest phylogenetic relatives, which explains the low number of species-specific proteins identified in both commercial products and their reference fruits (in-house extracts).

The following constraints were used for the searches: tryptic cleavage after Arg and Lys (semi-specific), up to two missed cleavage sites, tolerances of 20 ppm for precursor ions and 0.6 Da for MS/MS fragment ions, and allowance of optional Met oxidation and Cys carbamido-methylation. False discovery rates (FDRs) for peptide spectrum matches (PSM) and protein were limited to 0.01. Only those proteins with at least two unique peptides discovered from LC/MS/MS analyses were considered as reliably identified.

### 5.7. Statistics

One-way ANOVA was conducted to determine the significance of differences among sample results in protein quantification using Bradford assays, proteolytic assays, and stability tests. Post hoc analysis was performed using Tukey’s test in SigmaPlot 11 (Systat Software, San Jose, CA, USA), with significance set at *p* < 0.050 or <0.001.

## Figures and Tables

**Table 1:** Total identified proteins, proteolytic enzymes, and hypothetical protease content in fruit in-house extracts and commercial samples using proteomics.

Type of Sample	Total Identified Proteins ^**^	Proteolytic Enzymes ^***^	Percentage Ratio of Proteolytic Enzymes vs Total Identified Proteins
Kiwi Green *	503	11	2.2
K2	576	11	2
Kiwi Gold *	384	10	2.6
K5	749	10	1.3
Pineapple core *	471	10	2.1
Pineapple pulp *	515	14	2.7
P5	48	13	27
P4	14	6	42.9
Papaya *	35	6	17.1
PAP2	10	3	70
Fig *	21	8	38
F2	16	1	6.25

* Fruit in-house extracts. ** Proteins identified as different entries using a reference proteome or not depending on the database availability. *** Number of entries identified as full-length aspartyl and cysteine proteases (no fragments).

**Table 2:** LC-MS^2^-based proteomics identification of cysteine proteases and other main proteins previously observed by SDS-PAGE analyses.

Fruit	Database Entry (Uniprot)	−10log(P) ^a^	Coverage (%) ^b^	Protein Identification	Theoretical Mw (kDa) Based on Amino Acid or Nucleotide Sequence	Mr (kDa) SDS-PAGE
Kiwi Green/K2	P00785|ACTN_ACTCC **	455.00485/471.76282	42.4/53.4	Actinidin	27.5	26.5^•^
	A0A2R6QY36|A0A2R6QY36_ACTCC **	384.40567/420.12158	40.2/52.4	Actinidin-like	23.3	23.3^•^
	P83958|TLP_ACTCC **	383.77435/378.04047	52.9/52.9	Thaumatin-like protein	21.7	
	P85261|KIWEL_ACTCC **	452.24118/467.28903	77.9/74.2	Kiwellin	19.8	
	A0A2R6PEY1|A0A2R6PEY1_ACTCC **	290.15417/304.33725	46.3/37.9	KiTH-2 like	15.5	17.4^•^/14.0^•^
	A0A2R6PAW0|A0A2R6PAW0_ACTCC **	390.88022/391.0907	70.7/85.3	Kirola	17.2	
Kiwi Gold/K5	P00785|ACTN_ACTCC **	455.00485/399.8263	42.4/32.4	Actinidin	27.5	
	A0A2R6QY36|A0A2R6QY36_ACTCC **	384.40567/494.2946	40.2/62.2	Actinidin-like	23.3	22.8^•^
	P83958|TLP_ACTCC **	383.77435/268.69028	52.9/32.4	Thaumatin-like protein	21.7	20.3^•^
	P85261|KIWEL_ACTCC **	452.24118/504.32312	77.9/79.3	Kiwellin	19.8	
	A0A2R6PEY1|A0A2R6PEY1_ACTCC **	290.15417/289.5369	46.2/38.7	KiTH-2 like	15.5	13.4^•^
	A0A2R6PAW0|A0A2R6PAW0_ACTCC **	390.88022/432.87717	70.7/84	Kirola	17.2	
Fig/F2	A0A2Z6DRL4|A0A2Z6DRL4_FICCA	457.78674	59	Ficin 1a	23.6	22.9 ^†^/25.8 ^‡^
	A0A2Z6DRT1|A0A2Z6DRT1_FICCA	419.7921	57.6	Ficin 1b	23.6	
	A0A2Z6DRN1|A0A2Z6DRN1_FICCA	391.46454	36.1	Ficin 3	23.7	
	A0A2Z6DRM5|A0A2Z6DRM5_FICCA	245.10976	15.1	Ficin 2c	23.3	
	A0A2Z6DRL9|A0A2Z6DRL9_FICCA	245.10976	15.1	Ficin 2a	23.3	
	A0A2Z6DRP9|A0A2Z6DRP9_FICCA	245.10976	15.1	Ficin 2b	23.3	
	A0A182DW11|A0A182DW11_FICCA	239.45866	22.3	Ficin isoform D	23.4	
	A0A2Z6DRL5|A0A2Z6DRL5_FICCA**	448.26428/292.24368	51.1/34.7	Ficin 1c	23.6	
Papaya/PAP2	P14080|PAPA2_CARPA	367.02426	48	Chymopapain	23.7	23.8 ^‡^
	P05994|PAPA4_CARPA **	331.4425/ 363.13953	46.8/49.4	Papaya proteinase 4	23.3	
	P10056|PAPA3_CARPA **	271.31442/341.473	37.6/45.4	Caricain	23.4	
	H6USN0|H6USN0_CARPA	264.59772	25	Cysteine protease	ND	
	P00784|PAPA1_CARPA **	216.544/298.2124	28.4/43.2	Papain	23.4	
	H6USN1|H6USN1_CARPA	205.06474	16.3	Cysteine protease	23.4	
Pineapple core/pulp/P4/P5	A0A199W8R4|A0A199W8R4_ANACO ** (P4)	392.8472/125.80339/472.0772	18.9/5.9/29.7	Fruit bromelain (core)	ND	26.0^•^/23.0^•^ (core and pulp, and P5)/21.8 ^‡^/29.7 ^‡^ (P4)
	A0A199VSS3|A0A199VSS3_ANACO ** (P4 and P5)	339.10794/457.8168/228.36128/526.3434	32.9/47.5/33.7/54.5	Ananain (core and pulp)	22.9	
	A0A199UL32|A0A199UL32_ANACO	330.18286/ 428.22952	21.8/24.8	Ananain (core and pulp)	ND	
	A0A199W9F2|A0A199W9F2_ANACO ** (P4 and P5)	294.8118/452.54492/186.53676/540.68054	7.2/19.2/8.3/24.5	Fruit bromelain (core and pulp)	ND	
	O24641|O24641_ANACO	267.19427/285.8756	11.1/14.2	Bromelain (core and pulp)	22.9	
	A0A199W9E5|A0A199W9E5_ANACO ** (P4)	267.19427/285.8756/325.2002	11.1/14.3/15.7	Fruit bromelain (core and pulp)	22.9	
	A0A199UUY2|A0A199UUY2_ANACO ** (P4 and P5)	435.98737/164.08255/536.8819	40.7/24.5/68.3	Ananain (pulp)	ND	
	A0A199W8N4|A0A199W8N4_ANACO ** (P4 and P5)	412.75708/177.17471/451.46368	30.2/20.4/34.1	Ananain (pulp)	ND	
	A0A199UKV8|A0A199UKV8_ANACO ** (P4)	361.48822/434.87213	24.4/38.2	Ananain (pulp)	23.2	
	A0A199W3D2|A0A199W3D2_ANACO ** (P4 and P5)	425.63855/199.6339/534.271	14.1/11.1/23.1	Fruit bromelain (pulp)	ND	
	A0A199VJ20|A0A199VJ20_ANACO * (P5)	325.2002	15.7	Fruit bromelain	ND	
	A0A199UVV3|A0A199UVV3_ANACO * (P5)	158.96414	3.7	Fruit bromelain	21.9	

The absence of stars signifies only present in the fruit for proteomics analysis. * Protein identified in commercial products only by proteomics analysis. ** Protein identified in both in-house and commercial extracts by proteomics analysis. ^†^ Protein tentatively identified in the in-house extracts by SDS-PAGE. ^‡^ Protein tentatively identified in the commercial products by SDS-PAGE. ^•^ Protein identified in both in-house and commercial extracts by SDS-PAGE. ND, non-determined due to the presence of multiple domains in the nucleotide sequence. ^a^ Reliability of protein identification with the peptides detected grouped for that identification. The higher the value, the more reliable the identification. ^b^ Percentage of the sequence annotated in the database covered by the peptides identified in the samples.

**Table 3:** Description and labelling of tested commercial proteolytic products (PPs).

Sample	Fruit	Code *
Kiwi Green commercial products	*Actinidia deliciosa* (Kiwi Green)	K2, K3, K4, and K6
Kiwi Gold commercial products	*Actinidia chinensis* (Kiwi Gold)	K5
Papaya commercial products	*Carica papaya* (Papaya)	PAP1 and PAP2
Pineapple commercial products	*Ananas comosus* (Pineapple)	P2, P4, P5, and P6
Fig commercial product	*Ficus carica* (Fig)	F2

* The code is used to define the different commercial products of each type of fruit. The letter represents the initial or initials of the fruit’s name, while the number differentiates each product to maintain anonymity and confidentiality. K = kiwi; PAP = papaya; P = pineapple; F = fig.

## Data Availability

The raw data supporting the conclusions of this article will be made available by the authors on request.

## Supplementary Materials

The following supporting information can be downloaded at: https://www.mdpi.com/article/10.3390/ijms251910315/s1.
