# Supplementary material for: New Pipeline for Analysing Fruit Proteolytic Products Used as Digestive Health Nutraceuticals
Source: Int J Mol Sci. 2024 Sep 25;25(19):10315. doi: 10.3390/ijms251910315 (PMC11476805; doi:10.3390/ijms251910315)
Supplement: Supplementary file 1 [file ijms-25-10315-s001.zip › ijms-3169664-supplementary.pdf]

SUPPLEMENTARY MATERIAL

New pipeline for analysing fruit proteolytic products used as digestive health nutraceuticals

Iván Benito-Vázquez[1,2], Ana Muñoz-Labrador[1], Manuel Garrido-Romero[1,2], Gema Hontoria-Caballo[2], Carlos García-García[3], Marina Diez-Municio*[2], F. Javier Moreno*[1]

[1] Instituto de Investigación en Ciencias de la Alimentación, CIAL (CSIC-UAM), Nicolás Cabrera 9, 28049 Madrid, Spain

[2] Pharmactive Biotech Products SLU, Faraday 7, 28049 Madrid, Spain

[3] Centro de Biología Molecular Severo Ochoa, CBM (CSIC-UAM), Nicolás Cabrera, 1, 28049 Madrid, Spain

*Corresponding authors: javier.moreno@csic.es; mdiez@pharmactive.eu

Figure S1. Calibration curves to quantify proteolytic activity. A) Tyrosine (Tyr) calibration curve to calculate Casein Digestion Units (CDU). B) 4-nitrophenol calibration curve (4-NP) to enzymatic units' calculation from 0,04 - 0,3 µmol 4-NP and C) for 0,4 - 8 µmol 4-NP.

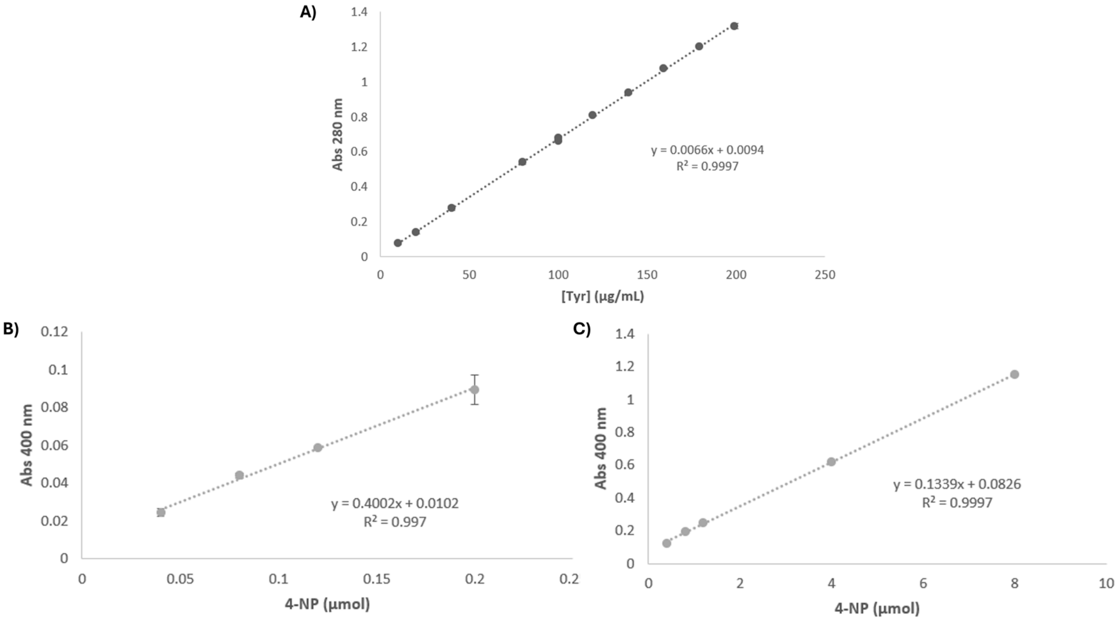

Figure S2. Stability test in long-term conditions (25 °C, 60 % relative humidity) using Z-L-Lys-ONp hydrochloride assay during 6 months for K2 (A), F2 (B), P4 (C), and PAP2 (D). T0, start. T1, 1 week. T2, 2 weeks. T3, 3 weeks. T4, 1 month. T5, 1.5 months. T6, 2 months. T7, 2.5 months. T8, 3 months. T9, 6 months. * p < 0.001, significant difference with respect to T0. Horizontal bar to compare bars at both ends.

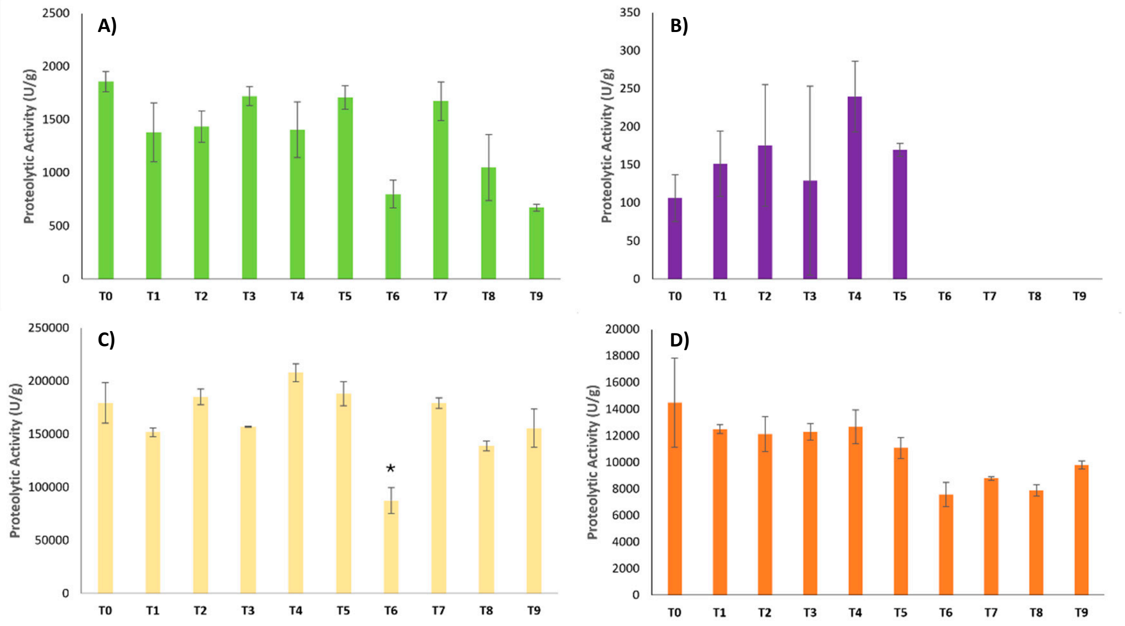

Figure S3. Comparison of A) *Ficus carica* ficin amino acid sequences and B) identity percentages. Multiple alignments were done using the program ClustalOmega. Residues that are identical (*), conserved (:) or semiconserved (.) in all sequences are indicated.

A)

```
Ficin_2a             MASLTSLSSLLLLLVLVLVAAADGSSFEESNPIRLVSDGLRDFHSQLLQVVGNTRHALSF    60
Ficin_2b             MASLTSLSSLL-LLVLVLVAAADGSSFEESNPIRLVSDGLRDFHSQLLQVVGNTRHALSF    59
Ficin_2c             MASLTSLSSLL-LLLLVLVAAADGSSFEEWNPIRLVSDGLRDFHSQLLQVVGNTRHALSF    59
Ficin_isoform_D      ------------------------------------------------------------    0
Ficin_3              -MDLTVFTTILT---IVVCLALS---YP---P-------------LTSSRTDVEVREMF    36
Ficin_1c             -MALTVFTTILT---IVLCLALS---YP---P-------------LTSSRTDVEVREMF    36
Ficin_1b             -MALTVFTTILT---IVLCLALS---YP---P-------------LTSSRTDAEVREMF    36
Ficin_1a             -MALTVFTTILT---IVLCLALS---YP---P-------------LTSSRTDAEVREMF    36


Ficin_2a             TRFASKYGKRYETEEEIKLRFEIFSENLKIIKSSNR-KGLPYTLAVNQFADWTWEEFKQH    119
Ficin_2b             TRFASKYGKRYETEEEIKLRFEIFSENLKIIKSSNR-KGLPYTLAVNQFADWTWEEFKQH    118
Ficin_2c             TRFASKYGKRYETEEEIKLRFEIFSENLKIIKSSNR-KGLPYTLAVNQFADWTWEEFKQH    118
Ficin_isoform_D      ------------------------------------------------------------    0
Ficin_3              VKWMATHGRAYNSLEEEERRFEIFKDNLRFVDEHNAIQNRTYKVGMNNFSDMTDEEYRRD    96
Ficin_1c             VNWMATHGRAYNSLGEEERRFEIFKDNLRFVDEHNAVQNRTYKVGMNMFADMTNEEYRRK    96
Ficin_1b             VNWMATHGRAYNSLGEEARRFEIFKDNLRFVDEHNAVQNRTYKVGMNTFADMTNEEYRRK    96
Ficin_1a             VNWMATHGRAYNSLGEEARRFEIFKDNLRFVDEHNAVQNRTYKVGMNTFADMTNEEYRRK    96


Ficin_2a             KLGAAQNCSATN-----KGSHKLTDVVLPKTKDWRETGIVSPVKNQGSCGSCWTFSTTGA    174
Ficin_2b             KLGAAQNCSATN-----KGSHKLTDVVLPKTKDWRETGIVSPVKNQGSCGSCWTFSTTGA    173
Ficin_2c             KLGAAQNCSATN-----KGSHKLTDVVLPKTKDWRETGIVSPVKNQGSCGSCWTFSTTGA    173
Ficin_isoform_D      ---------------------------LPETVDWRSKGAVDPIRDQGKCGSCWVFSVTVV    33
Ficin_3              MLGTRSDPEL-KTKVDSSRYAPHAAESLPETVDWRSKGAVNPIRNQGKCGSCWTFSTVAV    155
Ficin_1c             MLGARVDPELINTKVASSRYAPHAAESLPETVDWRIQGAVNPIRNQGRCGSCWAFSVVAV    156
Ficin_1b             MLGARVDPELIKTKVASSRYAPHAAESLPETVDWRIQGAVNPIRNQGRCGSCWAFSVVAV    156
Ficin_1a             MLGARVDPELIKTKVASSRYAPHAAESLPETVDWRIQGAVNPIRNQGRCGSCWAFSVVAV    156
                                                **:* ***  * *.*:::** *****.**.. .

Ficin_2a             LEAAYKQAFGKDISLSEQQLVDCAGAFNNFGCNGGLPSQAFEYIKYNGGLETEEAYPYTG    234
Ficin_2b             LEAAYKQAFGKDISLSEQQLVDCAGAFNNFGCNGGLPSQAFEYIKYNGGLETEEAYPYTG    233
Ficin_2c             LEAAYKQAFGKDISLSEQQLVDCAGAFNNFGCNGGLPSQAFEYIKYNGGLETEEAYPYTG    233
Ficin_isoform_D      VEGIVKIVTDTLPSLSEQELLDCAPSYKDLGCKGGWMDKAYDYIIKDRGITSESDYPYTA    93
Ficin_3              VEGITKIVTNKLPSLSEQQLLDCAPSYQNLGCKGGWMNRAYDYIIKNRGITSQSNYPYTA    215
Ficin_1c             VESITKIVTDELPSLSEQQLVDCAKSARDLGCRGGWMTKAYDYIIKNGGITSQSNYPYTA    216
Ficin_1b             VEGISKIVTDELPSLSEQQLVDCATSYKNLGCSGGWMTKAYDYIIKNGGISSQSNYPYTA    216
Ficin_1a             VEGITKIVTDELPSLSEQQLVDCATSYKNLGCSGGWMTKAYDYIIKNGGITSQSNYPYTA    216
                     :*.  * . .   *****:*:*** : .::** **   :*::**  : *: ::. ****.

Ficin_2a             KNGLCKFSAENVAVQVVKSV-NITLGAEDELKHAVGIVRPVSVAFQ-VVRGFHFYKDGVF    292
Ficin_2b             KNGLCKFSAENVAVQVVKSV-NITLGAEDELKHAVGIVRPVSVAFQ-VVRGFHFYKDGVF    291
Ficin_2c             KNGLCKFSAENVAVQVVKSV-NITLGAEDELKHAVGIVRPVSVAFQ-VVRGFHFYKDGVF    291
Ficin_isoform_D      RKGACDSRKATEVVATISSYEDVPADNEDALKKAVA-NQPVSVAIDASSAAVKEYSSGVF    152
Ficin_3              RKGQCNKAKASQTVATIDRYENVPRNNENALKKAVA-NQPVSVAIDASSKAVKQYKSGVF    274
Ficin_1c             RKGECNVTKASQTVATIDRYESVPRNNENALKKAVA-NQPVSVTIEAGGRAFQLYKSGVF    275
Ficin_1b             RKGECNKDLASQIVATIDSYEHVPRNNENALKKAVA-NQPVSVTIEAGGKAFQLYKSGVF    275
Ficin_1a             RKGECNKDLASQIVATIDSYEHVPRNNENALKNAVA-NQPVSVTIEAGGRAFQLYKSGVF    275
                     ::* *.    .  * .:.    :  . *: **:**.  :****:::    ..: *..***

Ficin_2a             TSDTCGSTSQDVNHAVLAVGYGVENGVPYWLIKNSWGADWGIDGYFRMELG----KNMCG    348
Ficin_2b             TSDTCGSTSQDVNHAVLAVGYGVENGVPYWLIKNSWGADWGIDGYFKMELG----KNMCG    347
Ficin_2c             TSDTCGSTSQDVNHAVLAVGYGVENGVPYWLIKNSWGADWGIDGYFKMELG----KNMCG    347
Ficin_isoform_D      VG----SCGVANDHAVVVVGYGTESGVNYWLVRNSWGTKWGEEGYMKLERDTAHPAGKCG    208
Ficin_3              TG----SCGTKLDHAVVVVGYGTQNGVDYWLVRNSWGTNWGEQGYMKLQRNVAHPAGKCG    330
Ficin_1c             TG----PCGTKLDHAVVAIGYGSENDVDYWLVRNSWG-NWGERGYIKIQRNVAEPAGKCG    330
Ficin_1b             TG----SCGTKLDHAVVAIGYGSENGKDYWLVRNSWGTNWGERGYIKLQRNVAEPTGKCG    331
Ficin_1a             TG----SCGTKLDHAVVAIGYGSENDVDYWLVRNSWGTNWGERGYIKLQRNVAEPTGKCG    331
                     ..      .   :***:.:*** :..  ***::**** .**  **:::  .     . **

Ficin_2a             VATCASYPIV----------------------   358
Ficin_2b             VATCASYPIV----------------------   357
Ficin_2c             VATCASYPIV----------------------   357
Ficin_isoform_D      IAMESTYPVKAC--------------------   220
Ficin_3              IAMQATYPVKKGKTSNKPYWAYEVDAEMVVVA   362
Ficin_1c             IAMHSTYPVKK--TSTKPYWAYEVDAEMVALA   360
Ficin_1b             IAMQSTYPVKK--TSTKPYWAYEVDAEMVAVA   361
Ficin_1a             IAMQSTYPVKK--TSTKPYWAYEVDAEMVAVA   361
                     :*  ::**:
```

B)

	Ficin_2a	Ficin_2b	Ficin_2c	Ficin_isoform_D	Ficin_3	Ficin_1c	Ficin_1b	Ficin_1a
Ficin_2a	100.00%	99.72%	99.16%	42.45%	38.11%	37.80%	37.99%	38.30%
Ficin_2b	99.72%	100.00%	99.44%	42.92%	38.53%	38.23%	38.41%	38.72%
Ficin_2c	99.16%	99.44%	100.00%	42.92%	38.53%	38.23%	38.41%	38.72%
Ficin_isoform_D	42.45%	42.92%	42.92%	100.00%	77.73%	70.18%	71.23%	71.23%
Ficin_3	38.11%	38.53%	38.53%	77.73%	100.00%	84.12%	84.72%	84.72%
Ficin_1c	37.80%	38.23%	38.23%	70.18%	84.12%	100.00%	92.78%	93.89%
Ficin_1b	37.99%	38.41%	38.41%	71.23%	84.72%	92.78%	100.00%	98.34%
Ficin_1a	38.30%	38.72%	38.72%	71.23%	84.72%	93.89%	98.34%	100.00%
